# Editorial: New trends in the treatment of mood disorders

**DOI:** 10.3389/fpsyg.2023.1357198

**Published:** 2024-01-09

**Authors:** Carmen Concerto, Andrea Aguglia, Fortunato Battaglia

**Affiliations:** ^1^Psychiatry Unit, Policlinico University Hospital “G.Rodolico-San Marco”, Catania, Italy; ^2^Department of Neuroscience, Rehabilitation, Ophthalmology, Genetics, Maternal and Child Health (DINOGMI), Section of Psychiatry, University of Genoa, Genoa, Italy; ^3^IRCCS Ospedale Policlinico San Martino, Genoa, Italy; ^4^Department of Medical Sciences, Hackensack Meridian School of Medicine, Nutley, NJ, United States; ^5^Department of Neurology, Hackensack Meridian School of Medicine, Nutley, NJ, United States

**Keywords:** mood disorders, conventional therapies, neuromodulation, major depressive disorder, tDCS

Mood disorders, such as major depressive disorder (MDD) and bipolar disorder (BD), are psychiatric conditions influenced by a combination of psychosocial, genetic, and epigenetic factors (Mineo et al., 2022). Despite notable advancements, a significant number of patients do not achieve complete responsiveness to conventional therapeutic strategies and may experience a chronic course. Existing effective approaches encompass a spectrum from psychotherapies to psychopharmacology to neuromodulation. The intricate origins of mood disorders, coupled with an incomplete understanding of their fundamental neurobiology, contribute to the challenges in their treatment. The exploration of alternative and innovative treatment options has been driven by the limitations and drawbacks of traditional treatments, alongside an increasing trend in morbidity and mortality (Paul and Potter, 2024). Different approaches are employed to bolster inadequate responses to traditional treatments. Lately, there has been a focus on investigating off-label augmentation agents and non-pharmacological interventions to enhance clinical response and mitigate the risk of relapse and recurrence. Literature indicates a growing adoption of augmentation strategies in treating mood disorders (Yan et al., 2022), incorporating the use of nutraceuticals or other compounds as supplementary therapies (Concerto et al., 2023). Additionally, there has been a resurgence in interest in exploring psychedelic treatments within experimental psychological settings (Kozak et al., 2023). Among the innovative treatment avenues, emerging neuromodulation methods have displayed significant potential in addressing patients with incomplete responses (Albert et al., 2015; Mutz et al., 2018; Nicoletti et al., 2023).

The identification of new treatment approaches holds significant importance for patients, clinicians, and researchers alike. This Research Topic seeks to compile original research and review articles delving into new strategies for mood disorder treatment, along with promising new findings in the realm of treatments. The Research Topic included a total of four studies.

In the first article, Borgogna and Aita explore the longstanding impact of the serotonin hypothesis on mental illness models, especially regarding depression. This hypothesis suggests that disruptions in the serotonin neural system are a fundamental biological cause of mood disorders, leading to widespread prescribing of selective serotonin reuptake inhibitors (SSRIs). However, concerns about the safety, effectiveness, and legitimacy of SSRIs have emerged, casting doubt on their therapeutic benefits. The article raises questions about how clinical psychologists will respond to the potential decline of the serotonin hypothesis. It suggests various approaches, including fostering awareness through discussions within the clinical psychology community, adjusting how SSRIs are discussed with clients, collaborating with prescribing physicians, and exploring integrative, process-based research models for understanding disorders. Ultimately, the article underscores the importance for clinical psychologists to actively participate in the ongoing debate about the validity and ethics of SSRIs as a treatment method. The goal is to reduce human suffering and advance more valid approaches to comprehending and treating affective disorders.

The article by Sun et al. examines postpartum depression (PPD), highlighting its intricate mix of physiological, emotional, and behavioral changes and its potential long-term effects on family relationships. The article explores the potential of transcranial direct current stimulation (tDCS) as an innovative and noninvasive therapeutic method for addressing PPD. It outlines a preliminary randomized controlled trial (RCT) protocol designed to inform a larger, multi-center study assessing the effectiveness and safety of tDCS in treating PPD. The primary target for stimulation is the left dorsolateral prefrontal cortex (dlPFC), a key area for central nervous system function. The proposed mechanisms of action involve enhancing dlPFC excitability, regulating neurotransmitters such as gamma-aminobutyric acid (GABA), dopamine (DA), and 5-hydroxytryptamine (5-HT), and impacting dorsal raphe nucleus (DRN) 5-HT neurons to alleviate depressive symptoms. The article references existing evidence supporting the effectiveness of tDCS for depression, including studies involving pregnant patients and meta-analyses. It acknowledges challenges such as subjective factors affecting outcomes, issues with electrode placement stability, and the need for optimization. The article suggests incorporating functional neuroimaging techniques and high-resolution modeling in future studies to enhance understanding and treatment outcomes. It underscores the importance of conducting larger RCTs, adhering to medical ethics and safety guidelines, implementing clear population screening, and increasing sample sizes to improve the assessment of treatment efficacy for PPD.

Brasso et al.'s study examines the clinical characteristics of MDD in two subgroups of patients affected by a Major Depressive Episode (MDE) before and after the pandemic. The aim is to identify variables significantly associated with post-lockdown hospitalizations. Results indicate that post-lockdown hospitalizations are linked to less frequent psychiatric follow-up and more severe MDE with increased suicidal ideation and psychotic features. Suicidal ideation is notably connected to post-lockdown admissions, suggesting the pandemic's role in fostering such thoughts. The study suggests that factors like limited access to health services, social isolation, and economic losses during lockdown contribute to symptom severity and heightened suicidal ideation. The presence of psychotic features in MDE is also associated with post-lockdown hospitalizations, potentially due to delayed treatment and pandemic-related social challenges. Furthermore, the study highlights an escalation in psychopharmacological treatment during post-lockdown hospitalizations, involving increased antidepressant dosage and augmentation therapies, likely influenced by a higher proportion of severe MDE cases. Acknowledging limitations, the study concludes that patients with MDD admitted post-lockdown exhibit greater MDE severity. This underscores the necessity for heightened attention, resources, and intensive treatments, with a specific focus on suicide prevention, particularly in emergency contexts like the COVID-19 pandemic.

The study by Vilalta-Lacarra et al. explores the connection between depressive phenotypes, antidepressant treatment, and the risk of mortality in a community-based sample. Key findings include the cognitive-emotional depressive phenotype being linked to increased cancer mortality in both genders, while the somatic depressive phenotype increases mortality for other causes, particularly in men. The impact of psychopharmacological treatments on male cancer patients' survival varies based on the treatment type. The research identifies diverse risk factors for non-cancer mortality in men and suggests different biological mechanisms based on depression phenotype. Depressive symptoms are associated with reduced treatment adherence in cancer patients. The role of antidepressant treatment in cancer development remains uncertain, with SSRIs showing increased mortality for both cancer and non-cancer causes in men. Notably, anxiolytic and hypnotic treatments appear to have a protective effect in male cancer patients. While acknowledging limitations, the study emphasizes the need for further research to comprehend the intricate relationship between depression, antidepressant treatment, and mortality risk in individuals with cancer.

The collection of articles presented here contributes significantly to the evolving field of psychiatry, particularly in the understanding and treatment of mood disorders. Moving forward, these studies advocate for continued discourse, collaborative efforts, and the pursuit of diverse research models to enhance the effectiveness and ethical considerations in the field of psychiatry, ensuring more tailored and comprehensive approaches to addressing mental health challenges. Future research should explore alternative treatments, refine diagnostic criteria, and consider contextual factors, ultimately striving to improve the wellbeing of individuals experiencing depressive disorders.

## Author contributions

CC: Conceptualization, Writing—original draft, Writing—review & editing. AA: Supervision, Writing—review & editing. FB: Supervision, Writing—review & editing.
